# A Direct Comparison of Different Measures for the Strength of Electrical Synapses

**DOI:** 10.3389/fncel.2019.00043

**Published:** 2019-02-12

**Authors:** Georg Welzel, Stefan Schuster

**Affiliations:** Department of Animal Physiology, University of Bayreuth, Bayreuth, Germany

**Keywords:** electrical synapse, gap junction conductance, electrical coupling, coupling coefficient, electrical synapse plasticity

## Abstract

During the last decades it became increasingly evident that electrical synapses are capable of activity-dependent plasticity. However, measuring the actual strength of electrical transmission remains difficult. Usually changes in coupling strength can only be inferred indirectly from measures such as the coupling coefficient and the coupling conductance. Because these are affected by both junctional and non-junctional conductance, plastic changes can potentially be due to both components. Furthermore, these techniques also require the blocking of chemical transmission, so that processes that involve crosstalk between chemical and electrical synapses will be suppressed. To directly examine the magnitude of errors that can occur, we use dual whole-cell current- and voltage-clamp recordings from the soma of the pair of easily accessible, electrically coupled Retzius cells in the leech to simultaneously determine coupling coefficients, coupling conductances and directly measured gap junctional currents. We present the first direct and comparative analysis of gap junction conductance using all three methods and analyze how each method would characterize the response of gap junctions to serotonin. The traditional coupling coefficients showed severe deficits in assessing the symmetry and strength of electrical synapses. These were reduced when coupling conductances were determined and were absent in the direct method. Additionally, both coupling coefficient and coupling conductance caused large and systematic errors in assessing the size and time course of the serotonin-induced changes of gap junctional currents. Most importantly, both measurements can easily be misinterpreted as implying long-term gap junctional plasticity, although the direct measurements confirm its absence. We thus show directly that coupling coefficients and coupling conductances can severely confound plastic changes in membrane and junctional conductance. Wherever possible, voltage clamp measurements should be chosen to accurately characterize the timing and strength of plasticity of electrical synapses. However, we also demonstrate that coupling coefficients can still yield a qualitatively correct picture when amended by independent measurements of the course of membrane resistance during the experiments.

## Introduction

**FIGURE 1:**
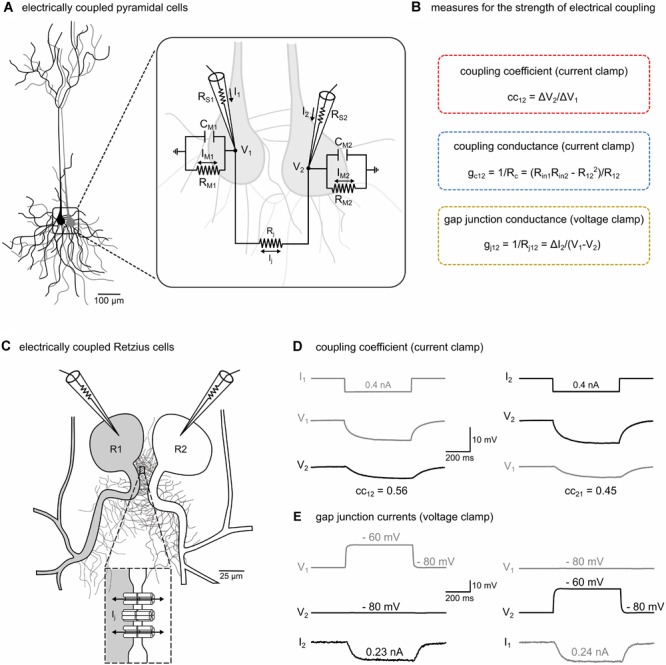
The strength of electrical transmission between electrically coupled neurons can be characterized in current-clamp and voltage-clamp mode. **(A)** Schematic representation of two typical central mammalian neurons (pyramidal cells) that are electrically coupled. The inset illustrates the equivalent circuit of the electrically coupled cells with recording microelectrodes. R_S1_, R_S2_: series resistances of microelectrodes. V_1_, V_2_: membrane potentials of the two cells. Cell membranes with resistances R_M1_, R_M2_ and capacitances C_M1_, C_M2_. R_j_: gap junctional resistance. I_j_: gap junctional current. I_1_, I_2_: currents sent through microelectrodes. I_M1_, I_M2_: currents across membrane resistances. **(B)** In central mammalian neurons, the strength of electrical transmission is typically inferred from indirect measures such as the coupling coefficient (cc) and the coupling conductance (g_c_) but not by directly measuring the gap junctional currents (I_j_) and conductance (g_j_). For details see section “Materials and Methods,” and **(D,E)**. **(C)** Schematic representation of the somata and the neurites emerging from the primary axons of a pair of Retzius neurons (R1 and R2) in the leech nervous system (adapted from Welzel and Schuster, 2018, with permission). The somata are electrically coupled by non-rectifying electrical synapses (see inset) between pairs of neurites within the neuropilar arborizations proximal to the soma (García-Pérez et al., 2004). **(D)** The coupled R cells allow the measurement and the direct comparison of all three methods. The cc was determined in each direction in current-clamp mode. A hyperpolarizing current (0.4 nA, 500 ms) was injected into one cell and the ratio of the voltage response of the non-injected cell (ΔV_2_) to that of the injected cell (ΔV_1_) was calculated. Additionally, input resistances (R_in_) and transfer resistances (R_12_ and R_21_) were calculated as needed to obtain estimates for g_c_. **(E)** To measure junctional currents, both cells were voltage-clamped at –80 mV. A probing brief depolarization to –60 mV (500 ms) applied to one cell is sufficient to detect the small junctional current (I_j_) from the change in current (ΔI_2_) in the clamp circuit of the other cell, directly yielding junctional resistance (R_j_) and conductance (g_j_ = 1/R_j_).

Electrical synapses formed by gap junction channels allow the direct flow of electrical currents between coupled neurons. Despite their simplicity, electrical synapses have been found to show a high degree of synaptic plasticity (Pereda et al., 2013; Curti and O’Brien, 2016; Haas et al., 2016). They are not only regulated by neuromodulators (Piccolino et al., 1984; Lasater and Dowling, 1985; McMahon et al., 1989; Pereda et al., 1992; Johnson et al., 1993; Smith and Pereda, 2003; Lefler et al., 2014; Mathy et al., 2014; Turecek et al., 2014; Wang et al., 2015) but also by activity-dependent mechanisms (Yang et al., 1990; Smith and Pereda, 2003; Landisman and Connors, 2005; Haas and Landisman, 2012b; Lefler et al., 2014; Mathy et al., 2014; Turecek et al., 2014; Wang et al., 2015). Several studies have demonstrated that neurotransmitter-dependent plasticity can be associated with the direct regulation of gap junction conductance (Lasater and Dowling, 1985; Lasater, 1987; DeVries and Schwartz, 1989; McMahon et al., 1989). However, most evidence is based on the indirect assessment of synaptic strength, typically by determining the so-called coupling coefficient (cc). This convenient measure characterizes the strength of electrical coupling of two cells by injecting a hyperpolarizing current pulse into one cell, and by measuring the associated changes in membrane potential in the injected (ΔV_1_) and in the other (non-injected) cell of the pair (ΔV_2_). The ratio between the changes in membrane potential of the two cells is then defined as the coupling coefficient (cc = ΔV_2_/ΔV_1_). However, the equivalent electrical circuit of an electrically coupled pair of cells (Figure 1A) shows, that the cc depends also on the resistance of the cell membrane. Specifically, changes in cc can in principle occur without any changes in electrical transmission at all, simply because of changes in membrane resistance. Hence, measuring coupling coefficients always bears some risk of not necessarily reflecting the strength of electrical synapses but of being confounded by non-junctional membrane properties of the coupled cells (Bennett, 1966; Pereda et al., 2013; Snipas et al., 2017). Plastic changes in non-junctional membrane plasticity can be a component of changes in cc and it is not easy to disentangle them from any plasticity of the electrical synapses. An important elaboration of the cc is the so-called coupling conductance (g_c_). This is calculated from cc values, taking estimates of the cells input resistances (R_in_) into account (Figure 1B). It is determined as appropriate for two isopotential cells connected by a single electrical synapse (Bennett, 1966) and will be less useful when deviations from these conditions occur. It also cannot completely disentangle junctional from non-junctional conductance changes, because the measurements of R_in_ at the soma that are needed to calculate g_c_ are also affected by changes in gap junctional resistance and can thus only serve as an estimate for the ‘true’ input resistance (Pereda et al., 2013). Thus, in most preparations, also g_c_ is likely to allow only a rough estimate of gap junctional strength (Bennett, 1966; Pereda et al., 2013; Shimizu and Stopfer, 2013; Curti and O’Brien, 2016). Nevertheless, both cc and g_c_ are generally used to demonstrate neurotransmitter-dependent or activity-dependent regulation of electrical synapses (Landisman and Connors, 2005; Haas et al., 2011; Haas and Landisman, 2012a; Mathy et al., 2014; Wang et al., 2015; Sevetson et al., 2017). In consequence, it is often not completely clear whether and to what extent plasticity of electrical coupling might also involve changes in the non-junctional membrane conductance. To suppress some of the non-junctional changes, cell membranes are often rendered passive by applying cocktails of antagonists and channel blockers (Landisman and Connors, 2005; Wang et al., 2015; Szoboszlay et al., 2016), an approach that clearly comes at the cost of also taking out potential signaling pathways that may be involved in the plasticity of electrical synapses.

In principle, it is possible to directly measure gap junction conductance (g_j_) in ways that are not confounded by other electrical parameters and do not require the blocking of chemical transmission. This approach uses coupled whole-cell voltage-clamp recordings from a pair of cells (Müller et al., 1999; Welzel and Schuster, 2018). However, the technique requires a working voltage clamp protocol, which limits the systems in which it could be used and will typically not be applicable in central mammalian neurons with large dendritic trees (Williams and Mitchell, 2008).

In order to explore the degree of possible limitations in the methods that we need to use in most mammalian neurons, we used a test-system that allowed us to simultaneously run all three methods on a given pair of coupled cells. This allowed us to directly compare how the three methods would interpret the effect of serotonin on gap junctions between these cells. This should be of great value to directly assess possible limitations of the most widely used methods of inferring plasticity in electrical synapses, and should lend confidence on how to detect and to correct them. For this approach, we used the pair of Retzius (R) cells in the nervous systems of the leech (*Hirudo medicinalis*). These cells can be voltage-clamped and allow the accurate measurement of gap junctional currents (Welzel and Schuster, 2018). They are coupled by electrical synapses between pairs of neurites in sufficiently close proximity (<50 μm) to the soma (García-Pérez et al., 2004) to avoid voltage clamp errors (Williams and Mitchell, 2008). By using dual whole-cell current- and voltage-clamp recordings from the soma of the coupled cells, we were able to experimentally determine in the same electrical synapse: (i) the coupling coefficient (cc), (ii) the estimated coupling conductance (g_c_), as well as (iii) a direct measurement of gap junction conductance (g_j_). We then compare the accuracy of the three methods in characterizing the time course of action of serotonin on the gap junctions. Our study thus presents the first direct and comparative analysis of all three methods available for characterizing electrical synapses and tests their suitability for a quantitative analysis of electrical synapse plasticity.

## Materials and Methods

### Leech Care

All experiments were performed with adult leeches (*Hirudo medicinalis*) from ANIMAL PHARMA GmbH (Weismain, Germany). Leeches were maintained at 18°C in 25 l water tanks.

### Preparation of Segmental Ganglia

The dissection of electrically coupled Retzius (R) neurons was conducted as previously described (Schlue and Deitmer, 1980; Welzel and Schuster, 2018). Briefly, leeches were anesthetized on ice cooled water for at least 10 min. Segmental ganglia were dissected and removed from the animal and pinned, ventral side up to a superfusion chamber coated with Sylgard (Dow Corning).

Dissection was carried out in leech Ringer solution composed of (in mM): NaCl, 115; KCl, 4; CaCl_2_, 1.8; MgCl_2_, 1.5; glucose, 10; tris-(hydroxymethyl)-aminomethane (Tris) maleate, 4.6; Tris base, 5.4 (all Sigma), buffered to pH 7.4. The ventral glial sheath covering the ganglia was opened with a fine microscissor to apply serotonin directly between the R cells. All experiments were done on R cells from midbody ganglia (ganglia 7 to 16).

### Electrophysiology

The Ringer solution was used in all electrophysiological measurements as external solution at room temperature (22 ± 2°C). Intracellular somatic current- and voltage-clamp recordings of the R cell pairs were performed by using two sharp glass microelectrodes (15–30 MΩ). The electrodes were pulled from borosilicate glass (GB100TF-10, Science Products) on a P-97 puller (Sutter Instrument) and backfilled with 3 M potassium acetate. The R cells could be unequivocally identified by their size and position within the ganglia. The microelectrodes were connected to two coupled discontinuous single electrode voltage-clamp (dSEVC) amplifiers (SEC-05X, npi electronic). Synchronizing two dSEVC amplifiers allows a precise and direct measurement of gap junctional conductance, independent of series and membrane resistances (Müller et al., 1999; Welzel and Schuster, 2018).

### Application of Serotonin

The focal application of serotonin (80 mM in dH_2_O, Sigma) was performed using broken glass microelectrodes (2–4 MΩ). The microelectrode was positioned between the R cell pairs under visual control. Serotonin was pressure-injected (20 psi; Picospritzer, Toohey Company, Fairfield, NJ) at a flow rate of 2–4 nl/s.

### Data Acquisition and Analysis

The dSEVC amplifiers were used in a master-slave configuration with the same, synchronized switching frequency (35 kHz) and the duty cycle was set to 1/4. All current and voltage recordings were low-pass filtered at 2 kHz. For a detailed description of the operational principles of dSEVC amplifiers, see Müller et al. (1999). Hum noise (50 Hz) was eliminated by a filter (Humbug, Quest Scientific). The signals were digitally sampled with at least 2 kHz (Micro1401, Cambridge Electronic Design), monitored by an oscilloscope (TDS 2004C, Tektronix) and recorded using the Spike2 software (Cambridge Electronic Design). All stimulation protocols were generated and delivered by a stimulus generator (Master-8, AMPI).

The coupling coefficient (cc) was calculated for both cells in both directions (cc_12_ and cc_21_) in current-clamp mode by injecting a hyperpolarizing current (0.4 nA, 500 ms) in one cell and calculating the ratio of the steady-state voltage response of the non-injected cell (ΔV_non-injected_) to that of the injected cell (ΔV_injected_):

(1) $$\begin{array}{c} \mathrm{cc}=\;\Delta V_{\mathrm{non}-\mathrm{injected}}/\Delta V_{\mathrm{injected}} \end{array}$$

The coupling conductance (g_c_) was estimated in each direction (g_c12_ and g_c21_) based on a model of two isopotential neurons and a single electrical junction following Bennett, 1966:

(2) $$\begin{array}{c} g_{c12}=\;{1/R}_{c12}=\left(R_{in1}R_{in2}-\;R_{12}^{2}\right){/R}_{12} \end{array}$$

where R_in1_ and R_in2_ represent the somatic input resistances of the coupled cells. R_in_ was defined as the voltage response of the injected cell divided by the amplitude of the injected current (0.4 nA, 500 ms). The transfer resistance (R_12_) was defined as the voltage response in R cell 2 when current was injected into R cell 1 divided by the amplitude of the injected current (0.4 nA, 500 ms).

We selectively measured the gap junctional currents (I_j_) between the coupled R cells as previously described (Welzel and Schuster, 2018). Briefly, we clamped both cells at a hyperpolarizing potential of -80 mV to inhibit the contribution of chemical synapses. A brief (200 ms) depolarizing voltage jump from -80 mV to -60 mV was induced in one cell (V_1_). The change in the current recorded from this cell (ΔI_1_) was the sum of the I_j_ and the membrane currents (I_M1_) in this cell. Because the other cell was continued to be clamped at -80 mV (V_2_), alterations of the current recorded in this cell (ΔI_2_) resulted only from the temporary voltage drop between the cells and was equal to -I_j_ (Figure 1E). The junctional resistance (R_j_) and conductance (g_j_) could thus be simply calculated from Ohm’s law:

(3) $$\begin{array}{c} g_{j12}=\;{1/R}_{j}=\Delta I_{2}/(V_{1}-\;V_{2}) \end{array}$$

To analyze the coupling symmetry, the R cells with the smaller cc, g_c_, or g_j_ were combined into one group (R cell 2; R2). The R cells with the larger cc, g_c_, or g_j_ were combined into another group (R cell 1; R1). The coupling symmetry was defined as the ratio of R2/R1 (cc_R2_/cc_R1_, g_c R2_/g_c R1_, or g_j R2_/g_j R1_). The cell with larger cc was not in each case the one with the larger g_c_ or g_j_, respectively.

**FIGURE 2:**
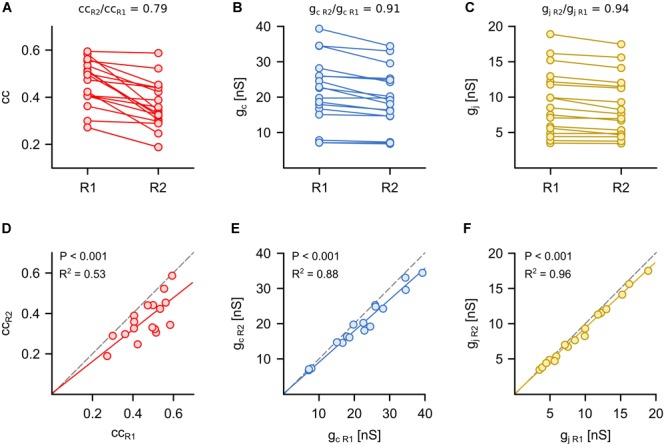
Symmetry of synaptic electrical transmission is best described by voltage-clamp based measurements of gap junction conductance. **(A)** The coupling coefficient (cc), **(B)** the coupling conductance (g_c_) and **(C)** the junctional conductance (g_j_) were determined in the same Retzius (R) cell pair in both directions (*n* = 17 pairs). The R cells with the smaller cc, g_c_, or g_j_ were combined into one group (R2). The R cells with the larger cc, g_c_, or g_j_ were combined into another group (R1). The mean coupling symmetry of each approach was defined as the ratio of respective values in R2 to R1. **(D–F)** Comparison of the smaller (R2) vs. the larger (R1) cc, g_c_, or g_j_ measured in both directions of coupling for each cell pair. Dashed lines indicate identical cc, g_c_, or g_j_ in both directions (slope = 1) and thus represent symmetrical coupling.

**FIGURE 3:**
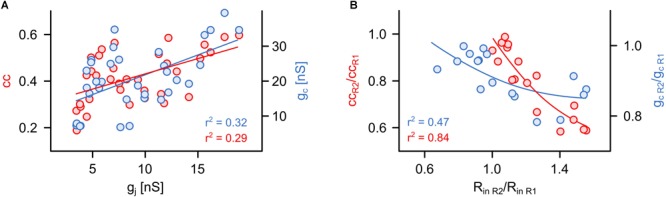
The coupling coefficient and the coupling conductance are strongly influenced by non-junctional factors. **(A)** Relationship between cc or g_c_ and simultaneously obtained actual value of g_j_ (*n* = 34 cells from 17 pairs). **(B)** Relationship between the apparent asymmetry of electrical coupling (cc or g_c_ ratios shown in Figure 2) and differences in input resistances (R_in_ ratio) of both R cells in each pair (*n* = 17 pairs).

Voltage deflections at the resistance of the microelectrodes were carefully compensated by means of the bridge balance controls of the dSEVC amplifiers. In each experiment the bridge balance was adjusted before and, if necessary, after penetrating a R cell by passing brief, hyperpolarizing current pulses (0.2 nA, 200 ms) until no instantaneous voltage change was observed at the beginning of the voltage response. Due to either slight under- or overcompensation of the bridge balance or to changes in the resistance of the microelectrode during the recordings, residual bridge imbalance errors can potentially occur. To ensure that the variability of the cc and the g_c_ (Figure 2, 3) did not result from such compensation errors, we used the high-resolution of the digitized data to determine any residual bridge imbalance errors in off-line analyses (Supplementary Figures S3, S4). First, we fitted the response of each R cell to hyperpolarizing current pulses with a double exponential function (Supplementary Figure S4A). A near instantaneous component at the onset of the voltage response (time constant τ_el_ < 1 ms) can be attributed to a voltage drop across the resistance of the electrode (Hewes and Truman, 1994). The slow component can be attributed to the cell membrane (membrane time constant τ_m_ = 60.4 ± 2.8 ms, *n* = 46). All recordings with τ_el_ < 1 ms thus indicate potential bridge imbalance errors and the effect of them on our pattern of results as well as any need for corrections (Hewes and Truman, 1994) can directly be analyzed (Supplementary Figures S4B,C). This analysis showed that all data could be included in the present study without any corrections needed.

Each R cell pair was only used for one experiment. We used only R cell pairs with stable membrane potentials and gap junctional currents (ΔI_2_) higher than 25 pA. All reported data of cc, g_c_, and g_j_ represent the average of at least 10 measurements. All data are reported as mean ± standard error of the mean (SEM). The normal distribution of variables was tested using the Shapiro-Wilk test. A Pearson correlation was used for linear regression analysis.

## Results

Most of the studies that analyzed the regulation and modulation of electrical synapses have been made in central neurons of the mammalian brain, e.g., pyramidal cells (Bennett and Zukin, 2004; Haas et al., 2016; Figure 1A). The plasticity in the strength of electrical transmission between electrically coupled neurons is usually inferred indirectly from the coupling coefficient (cc) and the coupling conductance (g_c_) (Figure 1B). Because these measures are also affected by changes in any of the other electrical parameters of neurons, e.g., the membrane resistances R_M1_ and R_M2_ of the coupled pair (Figure 1A), ideally voltage-clamp measurements of the junctional conductance (g_j_) should be used (Figure 1B). However, particularly in most mammalian central neurons, somatic voltage-clamp recordings are not able to accurately measure synaptic strength of dendritic gap junctions (Williams and Mitchell, 2008) and can therefore not be used as additional checks on the interpretations based on cc and g_c_. To directly assess limitations of using the simpler cc and g_c_ approaches, we took advantage of the pair of electrically coupled R cells in the leech (Figure 1C) that allows a direct measurement of g_j_ (Welzel and Schuster, 2018) and thus enables a direct comparison of all three methods (Figure 1D,E).

We began by comparing how each of the three methods assessed the symmetry of the electrical synapses, which is typically examined by using the coupling coefficient and the coupling conductance (Haas et al., 2011; Lefler et al., 2014; Wang et al., 2014). The R cells are electrically coupled by non-rectifying electrical synapses (Hagiwara and Morita, 1962) and provide an easy check of how suitable cc, g_c_ and g_j_ are to detect the expected symmetry in the coupling of the pair. For each R cell pair (*n* = 17), the strength of electrical coupling was determined in both directions (cc = 0.41 ± 0.02, g_c_ = 20.6 ± 1.5 nS, and g_j_ = 9.0 ± 0.8 nS; mean ± SEM, averaged between the two directions) and the neurons with the higher cc, g_c_, or g_j_ were defined as Retzius cell 1 (R1). Interestingly, cc_R1_ and cc_R2_ differed significantly (*P* < 0.001, Mann Whitney test) in 14 of the 17 pairs. Calculating the coupling symmetry based on coupling coefficients led to a value that was remarkably lower than unity (cc_R2_/cc_R1_ = 0.79 ± 0.03; Figure 2A), which would suggest that the coupling of R cells is typically not symmetrical (Figure 2D). Using g_c_ provides a different view. Here, the differences between g_c R1_ and g_c R2_ were smaller, which resulted in a symmetry closer to unity (g_c R2_/g_c R1_ = 0.91 ± 0.02; Figure 2B,E). This is far closer to indicating non-rectifying electrical coupling. The measurement of the gap junctional currents based on the voltage-clamp method clearly and directly showed a high degree of symmetry between g_j R1_ and g_j R2_ (g_j R2_/g_j R1_ = 0.94 ± 0.01; Figure 2C,F). We note that the assessment of symmetry was not statistically different between g_j_ and g_c_ but symmetry inferred from cc was significantly different (*P* < 0.01, one-way ANOVA) from both of them.

The assessment based on cc and g_c_ can indicate an asymmetric coupling of neurons not only in case of a rectifying conductance of the gap junction channels (Zolnik and Connors, 2016) but also when the input resistances (R_in_) differ between the pre- and postsynaptic cells (Bennett, 1966; Pereda et al., 2013; Snipas et al., 2017). As we know (and have seen by applying voltage-clamp measurements) that the junctional conductance between the R cells is not rectifying (Hagiwara and Morita, 1962), we directly analyzed to what degree cc and g_c_ actually depended on junction conductance g_j_ (as measured with the voltage-clamp method) as opposed to R_in_. Remarkably, both cc (*r*^2^ = 0.29; *P* < 0,001) and g_c_ (*r*^2^ = 0.32; P < 0,001) correlated only weakly with the actual gap junction conductance g_j_ (Figure 3A). Our findings suggest that the asymmetry inferred by using cc and g_c_ is related to differences of the input resistances R_in_ of the cells of a pair (Figure 3B). In summary, we show that the customary current clamp based methods fail to accurately report symmetry of an electrical synapse. In other words, any signs of rectification of an electrical synapse need to be checked very carefully, particularly when inferred from simple cc measurements. In our experiments, the measurements of cc and g_c_ depended only little on the (directly measured) actual gap junction conductance, but more on the input resistances. Thus, cc and g_c_ would erroneously suggest asymmetry of electrical coupling. Only with independent controls would it be possible to show that this interpretation is wrong and caused simply by a mismatch in input resistance.

**FIGURE 4:**
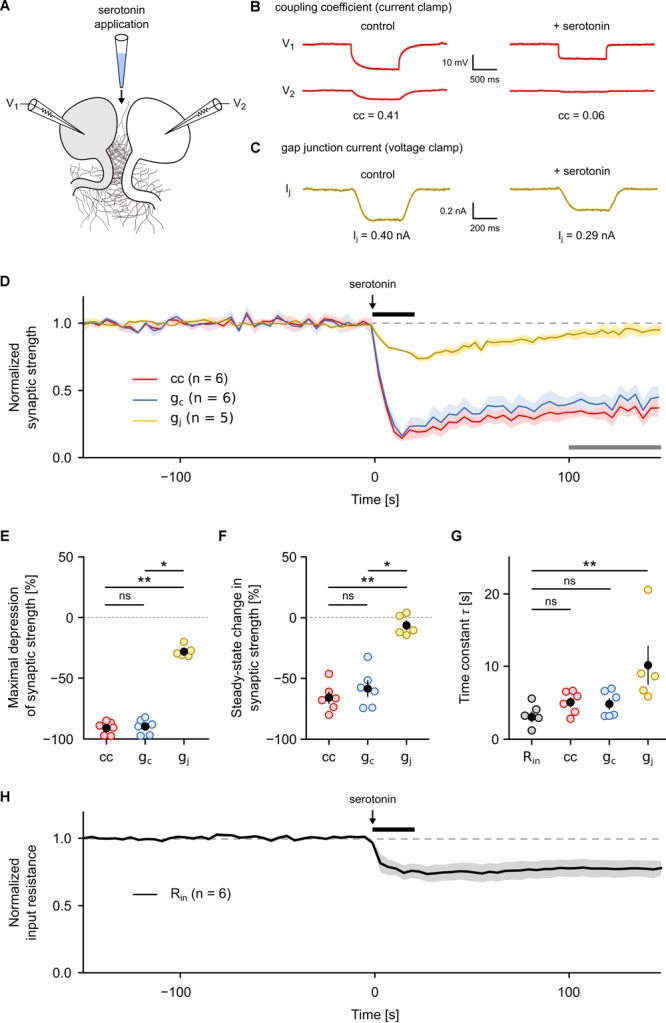
Gap junction conductance but not the coupling coefficient or the coupling conductance allows an accurate analysis of neurotransmitter-dependent plasticity of an electrical synapse. **(A)** Serotonin (80 mM) was pressure-injected locally with a micropipette placed between a R cell pair in which either the coupling coefficient (cc) and the coupling conductance (g_c_) or the gap junctional conductance (g_j_) was continuously monitored (0.25 Hz) in one direction as described (Figure 1). **(B)** Representative recordings illustrating the effect of serotonin application on cc and **(C)** on gap junctional currents. **(D)** Average traces of cc, g_c_, and g_j_ (normalized in each R cell pair to average pre-application level) showing a rapid depression of synaptic strength during application of serotonin for a duration of 20 s. Shaded areas represent SEM. Black horizontal line indicates the time of serotonin application. Gray horizontal line indicates the time interval used in **(F)** to calculate the steady-state long-term effect. **(E)** Maximal depression of cc (*n* = 6), g_c_ (*n* = 6), and g_j_ (*n* = 5) after serotonin application. **(F)** Long-term effect of serotonin application on cc (*n* = 6), g_c_ (*n* = 6), and g_j_ (*n* = 5) compared to the pre-application level. Open circles represent the mean cc, g_c_, and g_j_ during the last 50 s of the measurement period [indicated by the gray horizontal line in **(D)**]. **(G)** Time constant of serotonin action on R_in_ (*n* = 6), cc (*n* = 6), g_c_ (*n* = 6), and g_j_ (*n* = 5). Open circles show time constant derived from fitting the time course of the rapid depression phase (the first 30 s) in each individual trial. Black dots represent the mean value ± SEM. ^∗^*P* < 0.05; ^∗∗^*P* < 0.01; ns, not significant; Kruskal–Wallis test followed by Dunn’s *post hoc* test. **(H)** Average traces of R_in_ (normalized in each R cell pair to average pre-application level) showing a rapid and persistent decrease during application of a 20 s pulse of serotonin. Shaded areas represent SEM.

Our results show that both the cc and the g_c_ are less sensitive to actual gap junction conductance than they are to imbalances in other electrical properties, such as input resistance. Although this has long been suggested (Pereda et al., 2013; Shimizu and Stopfer, 2013; Curti and O’Brien, 2016), both cc and g_c_ are often our only ways to discover neurotransmitter-dependent or activity-dependent regulation of electrical synapses (Landisman and Connors, 2005; Haas et al., 2011; Haas and Landisman, 2012a; Mathy et al., 2014; Wang et al., 2015; Sevetson et al., 2017). Therefore, we asked whether and to what extent results on synapse plasticity would differ if they were inferred from current clamp approaches (i.e., from cc and g_c_). Since it is well established that the neuromodulator serotonin can reduce cc as well as R_in_ of the serotonergic R cells (Beck et al., 2002), we used the application of a pulse of serotonin to comparatively monitor the time course of its action with all three measures. For this purpose, we focally applied serotonin (80 mM in dH_2_O) for a duration of 20 s between the R cells (Figure 4A) and simultaneously measured the time course of cc and g_c_ (Figure 4B) as well as of g_j_ (Figure 4C) before, during, and after serotonin application. All three measures correctly detect the main effect of serotonin, a fast and strong reduction in gap junction conductance (Figure 4D). They also all showed that controls with only the solvent (dH_2_O) had no effect (Supplementary Figure S1). However, the differences in the actual time course, the strength of the effect and its longevity are striking. The maximal depression observed after serotonin application (Figure 4E) is much larger when assayed by cc (91.0 ± 2.2%; difference relative to baseline; *P* < 0.01) or g_c_ (89.9 ± 2.6%; difference relative to baseline; *P* < 0.05) whereas it appeared to be much smaller when measured with the voltage-clamp method that produced a significantly smaller drop by only 27.9 ± 2.1%. A further major discrepancy was in the plateau reached afterward. While g_j_ decayed to baseline, as expected due to the dilution of the short puff of serotonin and its diffusion into the bath, cc and g_c_ report an apparently persistent long-term effect (Figure 4F). During the last 50 s of the measurement period, the mean depression of both cc (65.7 ± 4.7%; *P* < 0.01) and g_c_ (58.3 ± 6.4%; *P* < 0.05) were significantly higher than g_j_ (6.2 ± 3.8%, Kruskal–Wallis test with Dunn’s *post hoc* test). The apparent persistence of an effect of serotonin on cc and g_c_ thus is neither consistent with the dilution (by a factor of 2.5–5.0 × 10^4^) nor is it compatible with the measurement of g_j_. The long-term component, seen in cc and g_c_ measurements, must therefore be interpreted very carefully and can certainly not be attributed to long-term changes in the electrical synapse itself. Not surprisingly, also details on the speed of action would be estimated differently by the three methods. The mean rate of decrease was much higher for cc (5.7% per second) or g_c_ (5.6% per second) compared to g_j_ (1.0% per second). Our measurements thus directly show that cc and g_c_ overestimated the size and speed of the effect of serotonin and, without further careful controls, could even be easily misinterpreted as indicative of a long-term effect on the electrical synapses. This does, however, not mean that the correct conclusion could not be reached based only on cc and g_c_, it only means that more care is needed. In our test case, measuring R_in_ of the cells (Figure 4H), as needed to infer g_c_, immediately detects changes in membrane conductance of the coupled cells. Moreover, the time constant of the changes in R_in_ (3.0 ± 0.7 s) after the onset of the serotonin pulse coincides roughly with that of the changes in cc (5.0 ± 0.6 s) and g_c_ (4.8 ± 0.7 s) but not with that of g_j_, which was significantly different (10.1 ± 2.7 s; *P* < 0.01; Kruskal–Wallis test with Dunn’s *post hoc* test) from that of R_in_ (Figure 4G and Supplementary Figure S2A). Moreover, the rapid depression of cc and g_c_ strongly correlates (*r*^2^ = 0.93; *P* < 0.001) with a similarly rapid depression of R_in_ (Supplementary Figure S2B). In summary, cc and g_c_ can produce results that can easily overestimate the size, the speed and longevity of effects of neuromodulators. Full reporting of possible changes of other electrical properties of the cells is therefore essential and is required to provide any hints at non-junctional effects. Wherever such additional controls are available, however, cc and g_c_ can be good methods to quantify the effect of neuromodulators on electrical synapses although they still might not allow a precise measurement of the time course of transmitter action.

## Discussion

The plasticity of electrical synapses is an exciting emerging field in neuroscience. Neurotransmitter- and use-dependent changes of the strength of electrical synapses are supposed to play important roles in brain function (Pereda et al., 2013; Haas et al., 2016). However, unequivocally quantifying plastic changes in the strength of electrical transmission is still difficult and plagued by methodological problems. The most widely used coupling coefficient (cc) does not directly measure junctional conductance but depends also on changes of membrane conductance and other electrical properties of neurons (Figure 1A). To reduce the effect of these other non-junctional effects, the coupling conductance (g_c_) was introduced that can be calculated based on experimentally measured input resistances and coupling coefficients (Bennett, 1966). However, the calculation of g_c_ is based on the assumption of two isopotential cells and is still more an estimate than a precise measure of the strength of electrical synapses (Pereda et al., 2013). Though these difficulties are known, actual measurements have been missing that would directly show the magnitude of the errors made. Our measurements show clearly that the errors are large and can by no means be neglected. By using a system that allowed us to simultaneously apply all currently available methods to monitor a serotonin-induced dynamic change, we directly demonstrate how sensitively cc and g_c_ can depend on non-junctional factors and how easily these measures can misinterpret the size, speed and even the longevity of a plastic change in gap junction conductance. We also demonstrated that even the symmetry of electrical coupling is difficult to assess by cc and g_c_ methods. Both measures correlated surprisingly weakly with actual gap junction conductance g_j_ and more with non-junctional properties.

Our findings also underscore how effective the use of suitable voltage clamp techniques would be. Recordings conducted with two discontinuous single-electrode voltage clamp amplifiers allow the precise and direct measurement of junctional currents and thus of conductance independently of membrane and series resistances (Müller et al., 1999). But, as previously discussed (Williams and Mitchell, 2008), somatic voltage clamp recordings are only able to accurately control voltage in somatic and proximal dendritic sites but not at distal dendritic sites of neurons (the so-called space-clamp problem). Additionally, the resistance and capacitance of dendritic membranes act as an electrical filter, leading to errors in the measurement of the junctional conductance in a distant-dependent manner. Even dendritic voltage-clamp recordings can be completely ineffective due to high spine neck resistance (Beaulieu-Laroche and Harnett, 2018). In the case of the electrical synapses between the coupled R cells, the close proximity of the gap junctions from the soma (García-Pérez et al., 2004) avoids large voltage-clamp errors due to space-clamp problems and this is supported by the ability of this method to detect the symmetry of the gap junction currents. The measurement of the gap junction currents thus allowed us to answer the unresolved issue whether serotonin indeed regulates the strength of the electrical synapses or only the non-junctional membrane conductance. In contrast to the experiments where we used cc and g_c_, we demonstrated a direct and fast regulation of g_j_ by serotonin that was independent of alterations in membrane resistances. The differences in the kinetics and the degree of depression revealed that the depression of cc and g_c_ was the sum of alterations in membrane and junctional resistance. Our measurements thus also show directly that measurements of cc together with that of the time-course of R_in_ (e.g., as in Haas et al., 2011) would arrive at the correct picture of gap junction conductance.

In summary, our results suggest that, if applicable, voltage clamp recordings should be used to characterize neurotransmitter- or use-dependent plasticity of electrical synapses instead of using the indirect cc or g_c_. This allows the direct measurement of gap junction currents independently of passive membrane properties and without the need of using substances to render the cell membranes passive. This might be particularly important for dissecting the molecular mechanisms of electrical synapse plasticity, where such substances might interfere with the underlying signaling pathways. Thus, the use of coupled cells in model systems that allow the direct measurement of gap junctional currents could push forward the identification of the molecular mechanisms of use-dependent plasticity of electrical synapses. The electrical synapses between the coupled R cells in the leech have already been shown to be capable of activity-dependent long-term potentiation by directly measuring gap junction currents (Welzel and Schuster, 2018) and are therefore a promising model. In addition, the high genetic amenability and the successful measurement of gap junctional currents between electrically coupled cells (Chen et al., 2007; Liu et al., 2017) would suggest that also *Caenorhabditis elegans* could be excellently suited for such an endeavor.

## Ethics Statement

According to the German Animal Protection law and approved by state councils (Regierung von Unterfranken, Würzburg, Germany) experiments on leech are exempt from any regulations.

## Author Contributions

GW performed and analyzed the experiments, and prepared the figures. GW and SS wrote the manuscript.

## Conflict of Interest Statement

The authors declare that the research was conducted in the absence of any commercial or financial relationships that could be construed as a potential conflict of interest.

## References

[B1] Beaulieu-Laroche L. Harnett M. T. (2018). Dendritic spines prevent synaptic voltage clamp. *Neuron* 97 75–82. 10.1016/j.neuron.2017.11.016 29249288

[B2] Beck A. Lohr C. Berthold H. Deitmer J. W. (2002). Calcium influx into dendrites of the leech Retzius neuron evoked by 5-hydroxytryptamine. *Cell Calcium* 31 137–149. 10.1054/ceca.2001.0268 12027387

[B3] Bennett M. V. L. (1966). Physiology of electronic junctions. *Ann. N. Y. Acad. Sci.* 137 509–539. 10.1111/j.1749-6632.1966.tb50178.x 5229812

[B4] Bennett M. V. L. Zukin R. S. (2004). Electrical coupling and neuronal synchronization in the mammalian brain. *Neuron* 41 495–511. 10.1016/S0896-6273(04)00043-1 14980200

[B5] Chen B. Liu Q. Ge Q. Xie J. Wang Z. W. (2007). UNC-1 regulates gap junctions important to locomotion in *C. elegans*. *Curr. Biol.* 17 1334–1339. 10.1016/j.cub.2007.06.060 17658257 PMC1976340

[B6] Curti S. O’Brien J. (2016). Characteristics and plasticity of electrical synaptic transmission. *BMC Cell Biol.* 17(Suppl. 1):13 10.1186/s12860-016-0091-y PMC4896238 27230893

[B7] DeVries S. H. Schwartz E. A. (1989). Modulation of an electrical synapse between solitary pairs of catfish horizontal cells by dopamine and second messengers. *J. Physiol.* 414 351–375. 10.1113/jphysiol.1989.sp017692 2558170 PMC1189146

[B8] García-Pérez E. Vargas-Caballero M. Velazquez-Ulloa N. Minzoni A. De-Miguel F. F. (2004). Synaptic integration in electrically coupled neurons. *Biophys. J.* 86 646–655. 10.1016/S0006-3495(04)74142-9 14695308 PMC1303833

[B9] Haas J. S. Greenwald C. M. Pereda A. E. (2016). Activity-dependent plasticity of electrical synapses: increasing evidence for its presence and functional roles in the mammalian brain. *BMC Cell Biol.* 17(Suppl. 1):14. 10.1186/s12860-016-0090-z 27230776 PMC4896267

[B10] Haas J. S. Landisman C. E. (2012a). Bursts modify electrical synaptic strength. *Brain Res.* 1487 140–149. 10.1016/j.brainres.2012.05.061 22771703 PMC3501583

[B11] Haas J. S. Landisman C. E. (2012b). State-dependent modulation of gap junction signaling by the persistent sodium current. *Front. Cell. Neurosci.* 5:31. 10.3389/fncel.2011.00031 22319469 PMC3263475

[B12] Haas J. S. Zavala B. Landisman C. E. (2011). Activity-dependent long-term depression of electrical synapses. *Science* 334 389–393. 10.1126/science.1207502 22021860 PMC10921920

[B13] Hagiwara S. Morita H. (1962). Electrotonic transmission between two nerve cells in leech ganglion. *J. Neurophysiol.* 25 721–731. 10.1152/jn.1962.25.6.721 13951916

[B14] Hewes R. Truman J. W. (1994). Steroid regulation of excitability in identified insect neurosecretory cells. *J. Neurosci.* 14 1812–1819. 10.1523/JNEUROSCI.14-03-01812.1994 8126573 PMC6577544

[B15] Johnson B. R. Peck J. H. Harris-Warrick R. M. (1993). Amine modulation of electrical coupling in the pyloric network of the lobster stomatogastric ganglion. *J. Comp. Physiol. A* 172 715–732. 10.1007/BF00195397 8350285

[B16] Landisman C. E. Connors B. W. (2005). Long-term modulation of electrical synapses in the mammalian thalamus. *Science* 310 1809–1813. 10.1126/science.1114655 16357260

[B17] Lasater E. M. (1987). Retinal horizontal cell gap junctional conductance is modulated by dopamine through a cyclic AMP-dependent protein kinase. *Proc. Natl. Acad. Sci. U.S.A.* 84 7319–7323. 10.1073/pnas.84.20.7319 2823257 PMC299284

[B18] Lasater E. M. Dowling J. E. (1985). Dopamine decreases conductance of the electrical junctions between cultured retinal horizontal cells. *Proc. Natl. Acad. Sci. U.S.A.* 82 3025–3029. 10.1073/pnas.82.9.3025 3857632 PMC397699

[B19] Lefler Y. Yarom Y. Uusisaari M. Y. (2014). Cerebellar inhibitory input to the inferior olive decreases electrical coupling and blocks subthreshold oscillations. *Neuron* 81 1389–1400. 10.1016/j.neuron.2014.02.032 24656256

[B20] Liu P. Chen B. Mailler R. Wang Z. W. (2017). Antidromic-rectifying gap junctions amplify chemical transmission at functionally mixed electrical-chemical synapses. *Nat. Commun.* 8:14818. 10.1038/ncomms14818 28317880 PMC5364397

[B21] Mathy A. Clark B. A. Häusser M. (2014). Synaptically induced long-term modulation of electrical coupling in the inferior olive. *Neuron* 81 1290–1296. 10.1016/j.neuron.2014.01.005 24656251 PMC3988996

[B22] McMahon D. G. Knapp A. G. Dowling J. E. (1989). Horizontal cell gap junctions: single-channel conductance and modulation by dopamine. *Proc. Natl. Acad. Sci. U.S.A.* 86 7639–7643. 10.1073/pnas.86.19.7639 2477845 PMC298122

[B23] Müller A. Lauven M. Berkels R. Dhein S. Polder H. R. Klaus W. (1999). Switched single-electrode voltage-clamp amplifiers allow precise measurement of gap junction conductance. *Am. J. Physiol.* 276 C980–C987. 10.1152/ajpcell.1999.276.4.C980 10199830

[B24] Pereda A. Triller A. Korn H. Faber D. S. (1992). Dopamine enhances both electrotonic coupling and chemical excitatory postsynaptic potentials at mixed synapses. *Proc. Natl. Acad. Sci. U.S.A.* 89 12088–12092. 10.1073/pnas.89.24.12088 1334556 PMC50703

[B25] Pereda A. E. Curti S. Hoge G. Cachope R. Flores C. E. Rash J. E. (2013). Gap junction-mediated electrical transmission: regulatory mechanisms and plasticity. *Biochim. Biophys. Acta Biomembr.* 1828 134–146. 10.1016/j.bbamem.2012.05.026 22659675 PMC3437247

[B26] Piccolino M. Neyton J. Gerschenfeld H. M. (1984). Decrease of gap junction permeability induced by dopamine and cyclic adenosine 3’:5’-monophosphate in horizontal cells of turtle retina. *J. Neurosci.* 4 2477–2488. 10.1523/JNEUROSCI.04-10-02477.1984 6092564 PMC6564702

[B27] Schlue W. R. Deitmer J. W. (1980). Extracellular potassium in neuropile and nerve cell body region of the leech central nervous system. *J. Exp. Biol.* 87 23–43. 7420016 10.1242/jeb.87.1.23

[B28] Sevetson J. Fittro S. Heckman E. Haas J. S. (2017). A calcium-dependent pathway underlies activity-dependent plasticity of electrical synapses in the thalamic reticular nucleus. *J. Physiol.* 595 4417–4430. 10.1113/JP274049 28369952 PMC5491877

[B29] Shimizu K. Stopfer M. (2013). Gap junctions. *Curr. Biol.* 23 R1026–R1031. 10.1016/j.cub.2013.10.067 24309273

[B30] Smith M. Pereda A. E. (2003). Chemical synaptic activity modulates nearby electrical synapses. *Proc. Natl. Acad. Sci. U.S.A.* 100 4849–4854. 10.1073/pnas.0734299100 12668761 PMC153644

[B31] Snipas M. Rimkute L. Kraujalis T. Maciunas K. Bukauskas F. F. (2017). Functional asymmetry and plasticity of electrical synapses interconnecting neurons through a 36-state model of gap junction channel gating. *PLoS Comput. Biol.* 13:e1005464. 10.1371/journal.pcbi.1005464 28384220 PMC5398722

[B32] Szoboszlay M. Lőrincz A. Lanore F. Vervaeke K. Silver R. A. Nusser Z. (2016). Functional properties of dendritic gap junctions in cerebellar Golgi cells. *Neuron* 90 1043–1056. 10.1016/j.neuron.2016.03.029 27133465 PMC4893164

[B33] Turecek J. Yuen G. S. Han V. Z. Zeng X.-H. Bayer K. U. Welsh J. P. (2014). NMDA receptor activation strengthens weak electrical coupling in mammalian brain. *Neuron* 81 1375–1388. 10.1016/j.neuron.2014.01.024 24656255 PMC4266555

[B34] Wang M.-H. Chen N. Wang J.-H. (2014). The coupling features of electrical synapses modulate neuronal synchrony in hypothalamic suprachiasmatic nucleus. *Brain Res.* 1550 9–17. 10.1016/j.brainres.2014.01.007 24440632

[B35] Wang Z. Neely R. Landisman C. E. (2015). Activation of group I and group II metabotropic glutamate receptors causes LTD and LTP of electrical synapses in the rat thalamic reticular nucleus. *J. Neurosci.* 35 7616–7625. 10.1523/JNEUROSCI.3688-14.2015 25972185 PMC6705436

[B36] Welzel G. Schuster S. (2018). Long-term potentiation in an innexin-based electrical synapse. *Sci. Rep.* 8:12579. 10.1038/s41598-018-30966-w 30135467 PMC6105662

[B37] Williams S. R. Mitchell S. J. (2008). Direct measurement of somatic voltage clamp errors in central neurons. *Nat. Neurosci.* 11 790–798. 10.1038/nn.2137 18552844

[B38] Yang X.-D. Korn H. Faber D. S. (1990). Long-term potentiation of electrotonic coupling at mixed synapses. *Nature* 348 542–545. 10.1038/348542a0 2174130

[B39] Zolnik T. A. Connors B. W. (2016). Electrical synapses and the development of inhibitory circuits in the thalamus. *J. Physiol.* 594 2579–2592. 10.1113/JP271880 26864476 PMC4865577

