# Supplementary material for: A Direct Comparison of Different Measures for the Strength of Electrical Synapses
Source: Front Cell Neurosci. 2019 Feb 12;13:43. doi: 10.3389/fncel.2019.00043 (PMC6379294; doi:10.3389/fncel.2019.00043)
Supplement: Supplementary file 1 [file Data_Sheet_1.docx]

Supplementary Material

**A Direct Comparison of Different Measures for the Strength of Electrical Synapses**

Georg Welzel & Stefan Schuster*

*** Correspondence:** stefan.schuster@uni-bayreuth.de


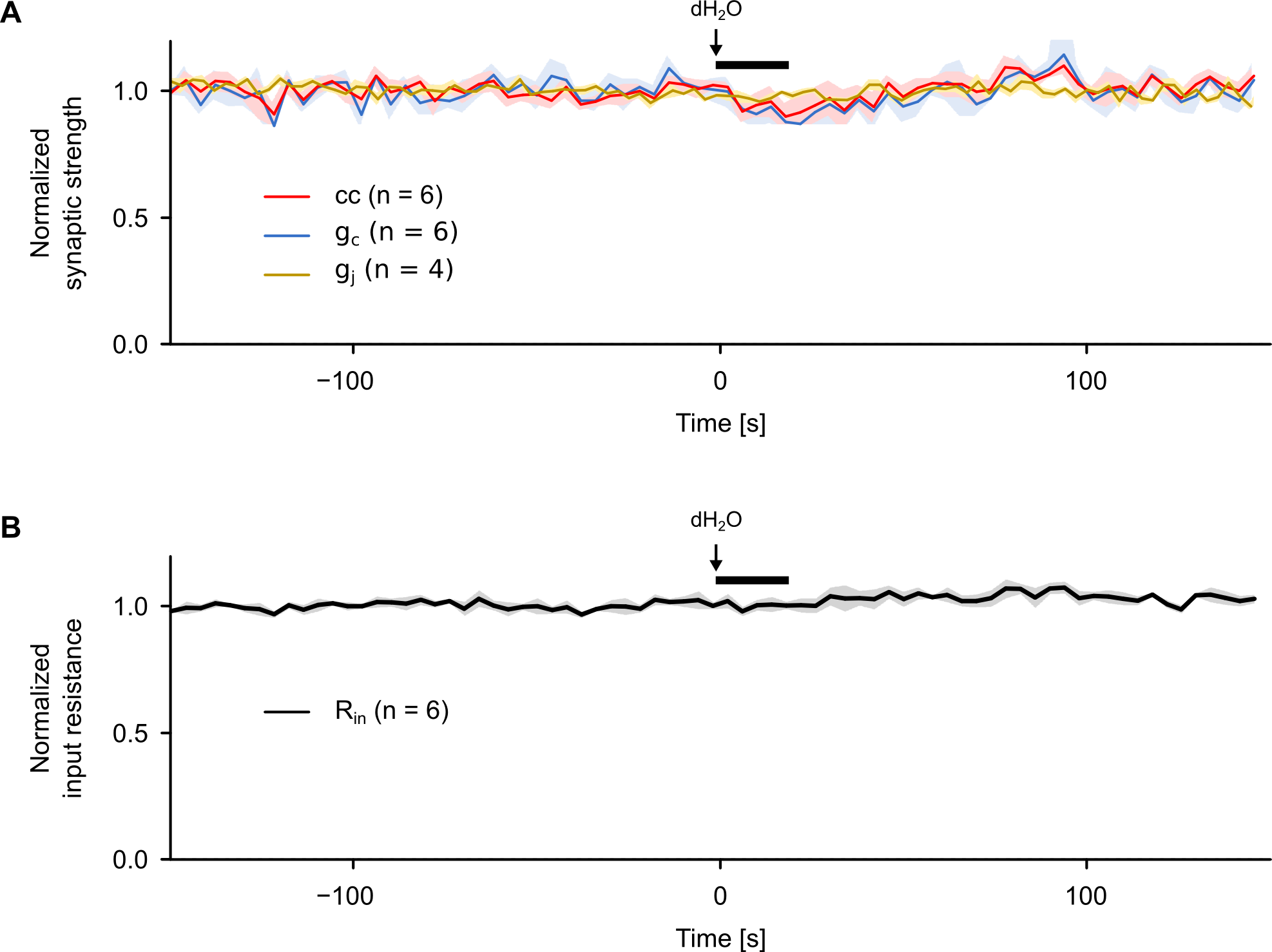


**Supplementary Figure 1.** Serotonin but not its solvent induced the depression of the synaptic strength. (**A**) Average traces of the coupling coefficient (cc), the coupling conductance (g_c_), the gap junction conductance (g_j_) and (**B**) the input resistance (R_in_) (normalized in each R cell pair to average pre-application level) showing no decrease of synaptic strength or input resistance during application of the solvent dH_2_O for a duration of 20 s. Shaded areas represent SEM. Black horizontal line indicates the time of dH_2_O application.


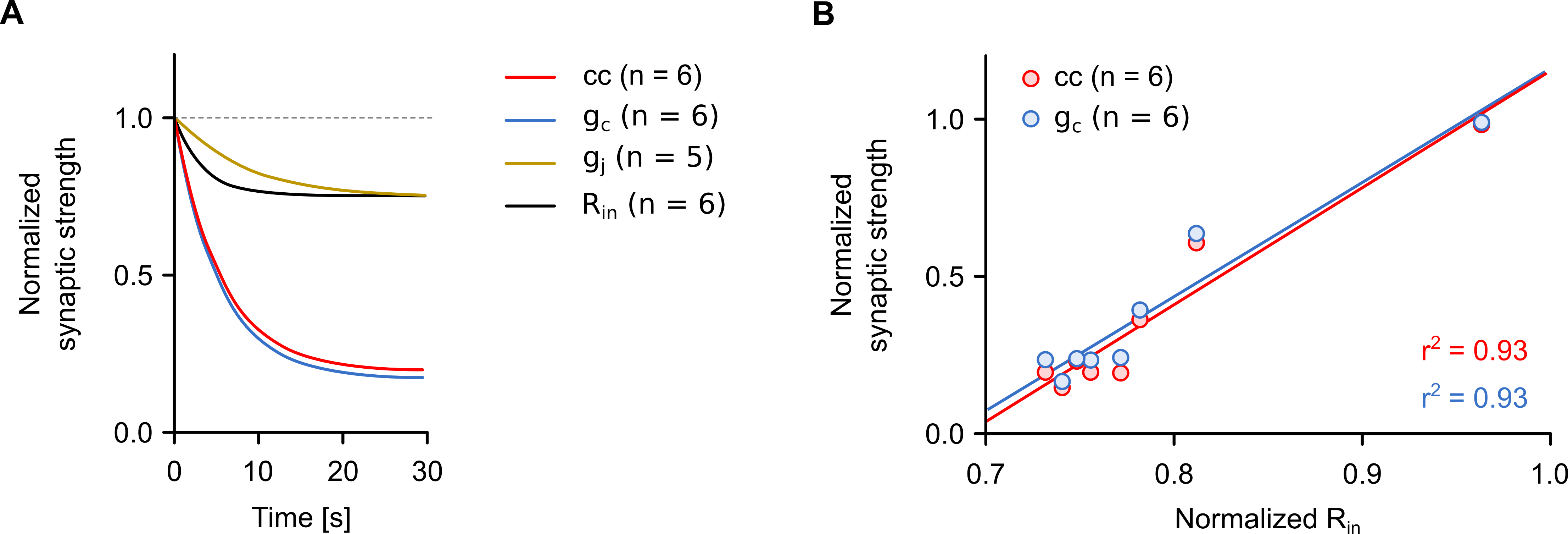


**Supplementary Figure 2.** Serotonin-dependent depression of the coupling coefficient or the coupling conductance correlates with the depression of the input resistance. (**A**) Curve fits of the initial depression of the coupling coefficient (cc), the coupling conductance (g_c_), the gap junctional conductance (g_j_) and the input resistance (R_in_) used to derive the time constant of serotonin action shown in Figure 4G. Each line represents the mean fit of the first 30 s after the beginning of serotonin application between the R cells. (**B**) Relationship between cc or g_c_, respectively, and R_in_ during the first 30 s after onset of serotonin application. Each circle represents the mean value (n = 6 R cells) at each time point. All values of cc, g_c_, g_j_ and R_in_ in (**A**) and (**B**) were normalized to the average pre-application level in each R cell pair.

**
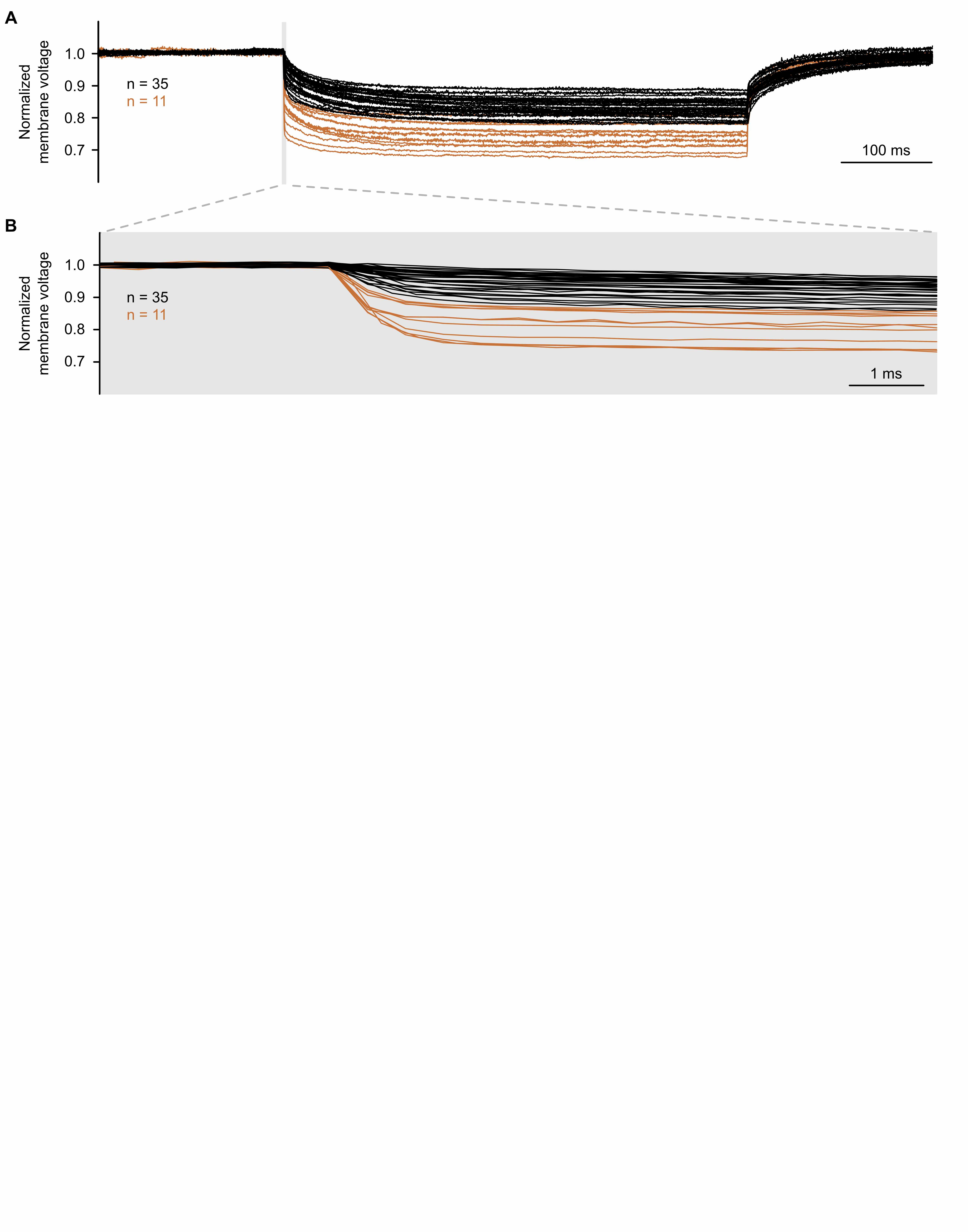
**

**Supplementary Figure 3.** Recordings to document potential errors in bridge balance for all experiments of the study. (A) Voltage drop to a hyperpolarizing current (0.4 nA, 500 ms). The initial voltage drop, highlighted in (B), typically (n = 35 R cells; black) shows no 'instantaneous' voltage drop (i.e. decay time constant τ_fast_ < 1 ms). Cases in which a rapid initial component was observed (brown) were used in the analysis of Supplementary Figure S4A to analyze whether our conclusions are robust against such small errors.

**
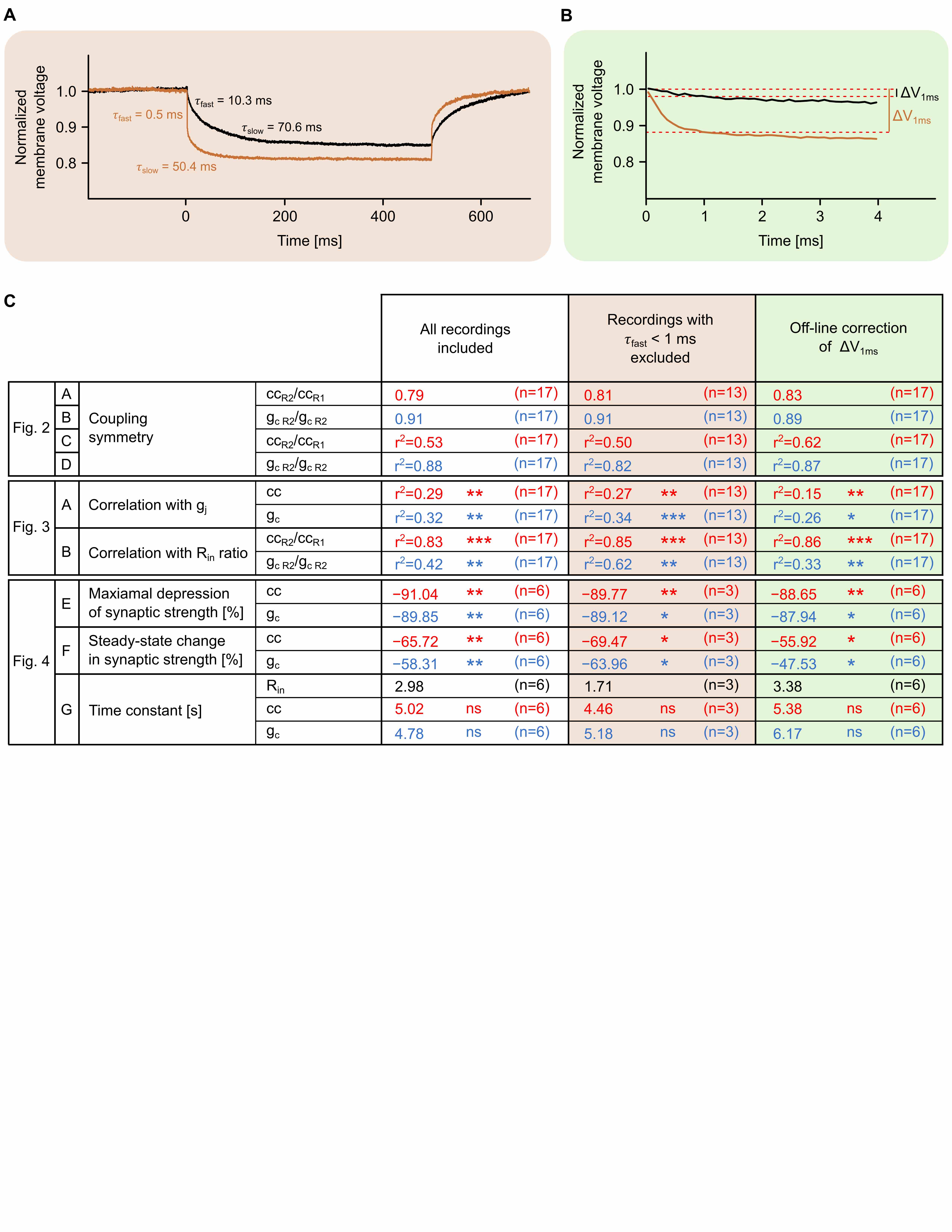
Supplementary Figure 4.** Analysis of the effect potential bridge balance errors could have on the results shown in Figures 2 to 4. (**A**) In the recordings of Supplementary Figure S3 the occurrence of an instantaneous voltage drop (τ_fast_ < 1 ms) at the onset (t = 0 ms) of the hyperpolarizing current step (0.4 nA, 500 ms) was detected by fitting the response of each R cells with a double exponential function. An instantaneous voltage response (τ_fast_ < 1 ms) can be attributed to a voltage drop across the resistance of the electrode (τ_fast_ = τ_el_). The slow component can be attributed to the cell membrane (τ_m_). This is illustrated with two normalized voltage traces, one with (brown) and one without (black) a residual bridge imbalance errors. A fast component was completely absent in 35 of the 46 traces shown in Supplementary Figure 3, a small one was detected in 11 of the 46 cases. (**B**) Potential voltage deflections across the electrode resistance can also be eliminated by subtracting voltage changes that occurred during the first millisecond (∆V_1ms_) from the total voltage deflection measured during the hyperpolarizing current step. (**C**) Results of re-analysis of all parameters of main Figures 2 to 4 when either the cases with detected small imperfections in bridge balance were excluded (brown background), based on the analysis shown in (A), or in which the correction shown in (B) was generally applied. The analysis shows that none of the conclusions depends on the occurrence of bridge balance errors.
